# Hypoxia-Independent Drivers of Melanoma Angiogenesis

**DOI:** 10.3389/fonc.2015.00102

**Published:** 2015-05-05

**Authors:** Svenja Meierjohann

**Affiliations:** ^1^Department of Physiological Chemistry, Biocenter, University of Würzburg, Würzburg, Germany; ^2^Comprehensive Cancer Center Mainfranken, University Hospital Würzburg, Würzburg, Germany

**Keywords:** melanoma, angiogenesis, hypoxia-independent, reactive oxygen species, NF-κB

## Abstract

Tumor angiogenesis is a process which is traditionally regarded as the tumor’s response to low nutrient supply occurring under hypoxic conditions. However, hypoxia is not a pre-requisite for angiogenesis. The fact that even single tumor cells or small tumor cell aggregates are capable of attracting blood vessels reveals the early metastatic capability of tumor cells. This review sheds light on the hypoxia-independent mechanisms of tumor angiogenesis in melanoma.

## Angiogenesis in Melanoma

Melanomas originate from cutaneous or extracutaneous melanocytes. In case of cutaneous melanoma, 25–35% of the tumors are derived from pre-existing nevi, although the origin of the remaining tumors is unknown. The transformation of nevi to melanoma can be reconstructed by a well-described progression model (1). By various oncogenic or epigenetic events, nevi can be stimulated to develop into the radial growth phase (RGP). At this stage, the pre-malignant cells expand in horizontal direction with restriction to the epidermis. RGP melanomas are <0.75 mm thick. When excised at this early stage, patients have an excellent prognosis, as the RGP tumors are generally not able to metastasize, and angiogenesis does not occur. During the next step, the transition to the vertical growth phase (VGP), melanomas gain the ability to cross the basal layer of the epidermis and to invade the deeper dermal layers of the skin. VGP tumors, which are >0.75 mm in size, have the potential to metastasize and to induce angiogenic events. The “angiogenic switch”, describing the gain of the ability to induce numerous pro-angiogenic factors, is a characteristic feature of the transition from RGP to VGP melanomas (2), and the pro-angiogenic features are even more enhanced in melanoma metastases. Pro-angiogenic factors can already be secreted by RGP melanoma cells *in vitro* under appropriate conditions, such as the presence of fibroblasts (3). However, because of their epidermal location, they are not able to attract blood vessels. Overall, the process of tumor angiogenesis contributes to the initial events of melanoma development, but it is also important for the maintenance, progression, and further spreading of already metastasized melanomas.

The diffusion limit of oxygen between cell and blood vessel is ~100–200 μm (4). Consequently, in absence of nutrient supply by the blood, the tumor size is limited to 0.2–2 mm in diameter (5). Interestingly, observations from animal models of melanoma show that tumor angiogenesis can already occur when melanomas are clearly below this size limit. In order to enable the detection of pro-tumorigenic events at this small-size scale, transgenic fish models with blood vessel-specific expression of fluorescent proteins are a valuable tool. In a xenotransplant model performed in zebrafishes which express eGFP under the *flk1* promoter (with *flk1* encoding Vegfr-2), it was shown that 15–30 injected melanoma cells already elicit an angiogenic response (6). For their experiments, the authors used the strongly metastatic murine B16-F10 melanoma cells. In our own laboratory, we investigated tumor angiogenesis in a genetic medaka fish melanoma model where the expression of a fish-specific oncogenic epidermal growth factor receptor (EGFR) leads to strong pro-tumorigenic signaling which is enhanced by autocrine loops (7, 8) and results in the malignant transformation of pigment cells and tumor development (9, 10). This fish EGFR is termed *Xiphophorus* melanoma receptor kinase (Xmrk) and was identified in naturally occurring melanoma of *Xiphophorus* fishes, the first described animal model for melanoma (11, 12). To visualize blood vessels, we used eGFP expression driven by the *fli-1* promoter, with Fli1 being a transcription factor strongly expressed in myeloid and endothelial cells. Strikingly, we observed that single melanoma cells had already the capacity to induce neoangiogenesis (13). These data are corroborated by earlier observations in a murine angiogenesis model of other tumor types based on dorsal skin-fold window imaging. Using fluorescent HCT116 human colon carcinoma cells and 4T1 murine mammary carcinoma, Cao and colleagues observed that incipient tumor angiogenesis can occur independently from hypoxia (14). In line with the results from these animal models, an association of angiogenesis with small cutaneous melanomas <1 mm in diameter was already described >20 years ago (15). To better comprehend the early steps of melanomagenesis, the following sections aim at increasing the understanding of angiogenic processes which can occur before a critical tumor mass is reached.

In general, tumor angiogenesis is regarded as a result from intratumoral hypoxia, which leads to the activation of hypoxia-inducible factor-1 (HIF1), one of the best-studied angiogenesis-related transcription factors. HIF1 is composed of the subunits HIF1α and HIF1β. HIF1β [also called aryl hydrocarbon receptor nuclear translocator (ARNT)] is constitutively expressed, whereas the abundance of HIF1α is strictly regulated and thereby determines the activity of the dimeric transcription factor. Under normoxic conditions, human HIF1α is hydroxylated on Pro402 and Pro564 by prolyl-4 hydroxylases (PDHs), thus leading to the recognition by the ubiquitin ligase component von Hippel Lindau protein (pVHL) and proteasomal degradation (16). PDHs exhibit an absolute requirement for oxygen, Fe^2+^ and α-ketoglutarate, the latter being a co-substrate for the enzymes. When pO_2_ is low, prolyl hydroxylation of HIF1α is suppressed, and the protein is stabilized (17). HIF1 is a potent inducer of pro-angiogenic factors including vascular endothelial growth factor (VEGF), angiopoietin-2, matrix metalloprotease 14 (MMP14), and angiogenin (18–20). Notably, next to angiogenesis, HIF1 regulates a large number of genes involved in invasion, differentiation, and metabolism as well as protection from apoptosis in melanoma. These processes are not in the scope of this review and are summarized elsewhere (21, 22).

Although HIF1α is already expressed in nevi, most likely as a result from the mild hypoxic conditions, which occur in skin (23), HIF1 expression and activity is increased in melanoma and correlates with decreased differentiation and VEGF expression (22, 24, 25). In a genetic mouse melanoma model driven by melanocyte-specific *Braf*^V600E^ induction and *Pten* depletion, HIF1α is required for angiogenesis and metastasis without affecting primary tumor formation (26).

## Hypoxia-Independent Regulation of HIF1α

In addition to the well-described hypoxia-dependent regulation mechanism, there are several hypoxia-independent processes, which can lead to the stabilization of HIF1α. These seem to play an important role in melanoma, where HIF1α is constitutively expressed – a fact, which is not necessarily due to the prevalence of hypoxic conditions. As an example, protein expression of HIF1α is strong in melanoma cells predominantly expressing HIF1α-785, a splice isoform lacking a crucial part of the oxygen-dependent degradation domain (27). Under normoxic conditions, HIF1α can be stabilized by various growth factors, cytokines and oncogenes, as shown for BRAF^V600E^ in melanoma (28). Next to BRAF/MEK/ERK signaling, the PI3K pathway is involved in the increase of HIF1α protein levels, e.g., via the PI3K downstream component 70 kDa ribosomal S6 kinase 1 (p70S6K1) (29–31). The PI3K pathway is frequently activated in melanomas because of deletions or inactivating mutations in the phosphatase PTEN or activation mechanisms, which encompass upstream components. In addition, endothelin-1 (ET-1)-dependent activation of the PI3K pathway could be directly linked to HIF1α stability under normoxic conditions in melanoma cells. Here, RNA and protein levels of the prolyl hydroxylase PHD2 were reduced in response to ET-1-dependent PI3K activation, and this was accompanied by increases in HIF1α (32). HIF1α accumulation can also take place in presence of enhanced reactive oxygen species (ROS) and NF-κB in melanoma (33, 34), both being commonly deregulated in this cancer type (35, 36).

The melanocytic master regulator and melanoma oncogene microphthalmia-associated transcription factor (MITF) is another important melanoma player. It was first described as a lineage-determining factor in melanocytes, which regulates the expression of genes involved in pigment production (37, 38). Nowadays, many more target genes of MITF are identified [reviewed in Ref. (39)], including *HIF1A*, which is regulated by MITF in a hypoxia-independent manner (40, 41). Notably, MITF cannot generally be considered as oncogenic, as its effects seem to depend on the expression level. While the entire depletion of MITF in melanoma cells can lead to cell cycle arrest and senescence, low MITF activity is associated with stem cell-like properties and invasiveness (42, 43). High MITF activity, however, promotes differentiation and exit from the cell cycle (44, 45). Different MITF levels lead to the targeting of different gene sets, which explains the observed concentration dependent effects. Next to the MITF expression level, protein modifications exhibit another measure to alter target gene expression. It could be demonstrated that the inheritance of mutant MITF^E318K^ predisposes to familial melanoma. This mutant exhibits defective SUMOylation, which results in an altered transcription of target gene subtypes in comparison to wildtype MITF. Among others, MITF^E318K^ shows enhanced binding to the HIF1α promoter and results in enhanced transcriptional activity of HIF1α (46).

However, HIF1α is not necessarily required for tumor angiogenesis. Early studies performed with RAS-transformed MEFs derived from *Hif1a*-/- mice showed that oncogenic HRAS is able to induce full tumor vascularization in the xenotransplants (47). Although HRAS plays a negligible role in melanoma, NRAS, which is activated in 15–20% of cutaneous melanoma, has strong functional overlap with the other RAS isoforms. These data are corroborated by the observation that mutant NRAS cooperates with the scaffold protein GAB2, which is often co-expressed with oncogenic NRAS, to increase angiogenesis and VEGF transcription in hypoxic and normoxic conditions in melanoma (48). By stimulating the expression of several matrix metalloproteases and urokinase plasminogen activator, RAS isoforms can furthermore contribute to the release and maturation of VEGF (49).

## Alternative Inducers of Hypoxia-Independent Angiogenesis

### Reactive oxygen species

Several inducers of cellular stress have the capacity to affect tumorigenic behavior by altering proliferative or metastasis-relevant effects.

As an example, reactive oxygen species (ROS) were reported to promote proliferation, migration and angiogenesis, if they are produced at sublethal concentrations. In endothelial cells, ROS, induced by NADPH oxidases, play an important role for proliferation, migration, and consequently the whole angiogenic sprouting process (50, 51). VEGFA and angiopoietin-1 belong to the factors, which activate endothelial NADPH oxidase activation (52–54). Consequently, tumors which secrete VEGFA or angiopoietin-1 lead to the production of pro-angiogenic ROS in the endothelial target cells. In addition, endogenously produced ROS inside the tumor trigger production and secretion of pro-angiogenic factors. Receptor tyrosine kinases, such as EGFR, are potent sources of intracellular ROS. The oncogenic fish EGFR Xmrk triggers the production of ROS in a manner, which includes the activation of NADPH oxidases. A high receptor density correlates positively with ROS levels and causes cellular senescence in melanocytes *in vitro* (55, 56). In developed melanomas of the Xmrk-expressing *Xiphophorus* or medaka fishes, several countermeasures are active to prevent the accumulation of toxic ROS levels [Ref. (57) and unpublished observations]. Still, slightly elevated ROS are required for hypoxia-independent angiogenesis, which can even be caused by single transformed cells (13). This was mediated by ROS-dependent activation of NF-κB, which led to the production of the pro-angiogenic factor angiogenin. Therefore, an early activation of NF-κB constitutes an exceptionally efficient angiogenesis trigger. The NF-κB subunit p50 correlates with a poor prognosis and reduced 5-year survival in melanoma and has been early implicated in angiogenesis (58, 59). Next to angiogenin and VEGF, IL-6 is another prominent example for a pro-angiogenic factor induced by NF-κB in melanoma (60).

NF-κB can be activated by several pathways in melanoma. It was described that the integrin-linked kinase ILK, which correlates with melanoma progression and invasion, enhances the activity of NF-κB and thereby leads to the secretion of IL-6 and VEGF in melanoma cells. As a consequence, microvessel formation is induced (61). In addition, NF-κB is suppressed by the breast cancer metastasis suppressor 1 (BRMS1), which effectively blocks angiogenic events. In melanoma, BRMS1 is decreased, and low expression is significantly correlated to reduced 5-year survival (62).

The ROS-induced DNA damage leads to the activation of DNA damage pathways. The serine/threonine protein kinase ataxia-telangiectasia mutated (ATM) is an important DNA damage sensor and inducer of checkpoints, which helps to halt the cell cycle to allow for DNA repair. P53, histone 2AX, and checkpoint homolog CHK2 belong to the protein targets of ATM. They are activated by phosphorylation, thus enabling them to fulfill their tumor-suppressive functions. However, ATM can also exert pro-angiogenic and tumor-promoting effects. In transplanted B16 melanomas, ROS led to the activation of endothelial ATM, which specifically resulted in tumor angiogenesis (63).

Furthermore, the nuclear factor (erythroid-derived 2)-like 2 (NRF2) is a transcription factor, which is strictly controlled by ROS levels. When cellular ROS stress is low, NRF2 is kept in the cytoplasm by its interaction partner, Kelch like-ECH-Associated Protein 1 (KEAP1), becomes ubiquitinated, and is quickly degraded. Under conditions of high reactive oxygen stress, critical cysteine residues of KEAP1 are disrupted, allowing for NRF2 stabilization and translocation to the nucleus. Here, NRF2 induces the transcription of genes involved in antioxidant pathways including the glutathione and thioredoxin pathways (64).

NRF2 is also indirectly involved in increasing the levels of VEGFA in an activating transcription factor 4 (ATF4) or HIF1α-dependent manner (65–69).

Although hypoxia and elevated ROS levels seem to contradict each other at first sight, they are strongly intertwined, and in solid tumors it is hardly possible to clearly distinguish between hypoxia-induced and ROS-induced angiogenesis. ROS are paradoxically generated under hypoxic conditions. This is presumably caused by reduced electron flux through the electron transport chain, which might lead to a prolonged lifetime of the semiquinone radical. This, in turn, might favor the generation of superoxide from molecular oxygen (70). The thus generated ROS can also contribute to HIF1α stability. Recently, Calvani and colleagues described that HIF1α enhances its own stability via a bicyclic ROS-mediated positive feedback loop in melanoma cells: during hypoxia, ROS are generated in the mitochondrial electron transport chain and elevate HIF1α stability, thereby leading to enhanced *VEGFA* expression. The following autocrine stimulation of VEGFR2 is further supporting HIF1α stabilization, presumably by a mechanism involving NADPH oxidase dependent ROS (71).

In addition to inducing angiogenic sprouting from existing blood vessels, some metastatic tumors are capable of vascular mimikry (VM). This observation was first made in aggressive melanomas (72) and was only later detected in other tumor entities. VM describes the transdifferentiation to an endothelial phenotype, thus allowing the formation of vessel-like structures and helping to maintain nutrient supply in the tumor. These structures can connect with the capillary network and are associated with poor prognosis (73). ROS seem to be important promoters of VM, as several ROS scavengers including *N*-acetylcysteine and resveratol strongly inhibit the formation of capillary structures in melanoma cells *in vitro* and, as demonstrated for resveratol, *in vivo* (74).

In addition to endogenous ROS production, cells of the tumor niche can generate various ROS species and thereby contribute to tumor angiogenesis. As an example, tumor-promoting macrophages are recruited to tumor cells by chemotactic factors including CCL2, VEGFA, and M-CSF. Macrophages are effective producers of ROS and reactive nitrogen species (RNS) (such as nitric oxide) and thus help to create a pro-inflammatory environment, which supports tumor angiogenesis (75).

Consistent with the role of ROS in melanoma progression and angiogenesis, the supplementation with antioxidants can prevent or alleviate the development of metastases. Selenium is an essential trace element needed for the generation of selenocysteine, an amino acid with high redox activity, which is present at catalytically active sites of various antioxidant enzymes. Selenite supplementation decreases the ROS load in cancer cells, with corresponding inhibitory effects on HIF1α, VEGF, and lung metastasis of murine melanoma cells (76, 77). The antioxidant *N*-acetylcysteine has similar anti-angiogenic as well as anti-metastatic effects (78). Owing to their numerous effects on signal transduction in and between cells, ROS and RNS are among the most efficient multi-functional angiogenesis and metastasis modulators.

## Unfolded Protein Response

Next to harboring the equipment for protein synthesis and maturation, the endoplasmatic reticulum (ER) is a crucial stress sensor and a regulator of cellular homeostasis. Nutrient starvation as well as nutrient excess are received as ER stress and are translated into different cellular responses which protect the cellular or tissue integrity. Owing to their high proliferation rate and altered metabolism, the demand for protein production in the ER is very high in tumor cells, thus generating conditions, which permit ER stress (79). The unfolded protein response (UPR) is a mechanism activated in the ER by the accumulation of mis- or unfolded protein, which can occur in fast growing tumors or under conditions of hypoxia, low pH, or low nutrient supply. The UPR generally serves the cellular integrity by enabling ER homeostasis under ER stress and is composed of three pathways, which stimulate protein folding, protein degradation, and the transient inhibition of protein synthesis (80). UPR activity also plays a role during tumor development, and the angiogenic switch was reported to be induced by the PERK/ATF4 pathway in a HIF1α independent manner (81). UPR activation is able to enhance the transcription of pro-angiogenic factors including VEGFA, FGF2, angiogenin, and angiopoietin-2 and to downregulate the expression of several angiogenesis inhibitors (81, 82). In melanoma, activation of the UPR component GRP78 is correlated with metastases and shorter survival (83).

Notably, the UPR can also be considered as a part of the ROS network: On the one hand, ROS can trigger the UPR and on the other hand, all three UPR pathways are implicated in the generation of ROS (79).

## PGC-1α

Observations from neoangiogenic processes of normal healthy tissues also provide valuable information, which can be relevant for tumor biology. In adipose tissue, hypoxia-independent angiogenesis during cold acclimation is regulated by mitochondrial proteins (84). Adipose tissue is prone to constant changes of size and volume and is accompanied by corresponding adaptations of tissue vascularization. When the organism is exposed to cold temperature, expression of the mitochondrial biogenesis regulator PGC-1α is induced. PGC-1α is able to activate *VEGFA* expression at the transcriptional level in an HIF1α-independent manner (85). Consequently, several pro-angiogenic factors including VEGFA are produced hypoxia independently by cold acclimation, leading to VEGFR2-dependent angiogenesis of the adipose tissue (84). A high expression of PGC-1α is found in ~10% of melanomas and is associated with poor overall survival (86). The link between PGC-1α, VEGF, and angiogenesis has not been investigated in melanoma to date. It would be interesting to analyze whether the survival disadvantage of patients with highly PGC-1α expressing melanoma is related to an increased tumor angiogenesis. Of note, BRAF inhibitors, which are routinely used in the clinic for the treatment of patients with BRAF^V600E^ positive melanomas, have been reported to suppress PGC-1α expression (87). Whether PGC-1α is re-expressed in recurrent melanoma and contributes to the fast progression after BRAF inhibitor resistance development is currently unknown. Table 1 summarizes the mentioned hypoxia-independent pro-angiogenic mechanisms in melanoma.

**Table 1:** **List of the hypoxia-independent pro-angiogenic mechanisms with relevance to melanoma**.

Angiogenesis inducer	Pro-angiogenic effect	Reference
BRAF^V600E^	*HIF1A* expression	(28)
PI3 kinase	Increase of HIF1 α protein	(29–31)
ET-1	Increase of HIF1 α protein	(32)
ROS	Increase of HIF1 α protein	(34, 71)
NF-κB	Increase of HIF 1 α protein	(33, 34)
MITF	*HIF1A* expression	(40, 46)
NRAS (with GAB2)	*VEGFA* expression	(48)
ILK	NF-κB activation, *IL-6*, and *VEGFA* expression	(61)
Reduction of BRMS1	Increased NF-κB activity	(62)
ROS	NF-κB activation, ANG expression	(13)
ROS	ATM induction	(63)
NRF2	*VEGFA* expression (ATF4- or HIF1α-dependent)	(65–69)
UPR	*VEGFA*, *FGF2*, *ANG*, *ANGPT2* expression	(81, 82)
PGClα	*VEGFA* expression	(84)

## Concluding Remarks

Pro-angiogenic events caused by hypoxia-independent cues allow incipient tumors to recruit blood vessels even before a critical tumor mass is reached. This provides the first entry point into the vasculature and might enable small tumor cell colonies, which produce the appropriate signals, to metastasize. Genomic information from several melanomas from different stages in the same patients lead to the conclusion that the first step of metastasis can occur very early during tumor formation (88, 89). The underlying reasons are most likely found in the incipient tumor-initiated angiogenesis of blood and lymph vessels. Although the colonization at distant sites is also determined by additional factors, the knowledge of the first possible metastatic events is invaluable to understand the timing of melanoma development.

## Author Contributions

SM reviewed the literature and wrote the article.

## Conflict of Interest Statement

The author declares that the research was conducted in the absence of any commercial or financial relationships that could be construed as a potential conflict of interest.

## References

[1] Zaidi MR Day CP Merlino G. From UVs to metastases: modeling melanoma initiation and progression in the mouse. J Invest Dermatol (2008) 128:2381–91. 10.1038/jid.2008.177 18787547

[2] Ria R Reale A Castrovilli A Mangialardi G Dammacco F Ribatti D Angiogenesis and progression in human melanoma. Dermatol Res Pract (2010) 2010:185687. 10.1155/2010/185687 20631829 PMC2901609

[3] Goldstein LJ Chen H Bauer RJ Bauer SM Velazquez OC. Normal human fibroblasts enable melanoma cells to induce angiogenesis in type I collagen. Surgery (2005) 138:439–49. 10.1016/j.surg.2005.06.031 16213896

[4] Schumacker PT Samsel RW. Analysis of oxygen delivery and uptake relationships in the Krogh tissue model. J Appl Physiol (1985) (1989) 67:1234–44. 2793716 10.1152/jappl.1989.67.3.1234

[5] Gasparini G. The rationale and future potential of angiogenesis inhibitors in neoplasia. Drugs (1999) 58:17–38. 10.2165/00003495-199958010-00003 10439927

[6] Zhao C Yang H Shi H Wang X Chen X Yuan Y Distinct contributions of angiogenesis and vascular co-option during the initiation of primary microtumors and micrometastases. Carcinogenesis (2011) 32:1143–50. 10.1093/carcin/bgr076 21515914

[7] Laisney JA Braasch I Walter RB Meierjohann S Schartl M. Lineage-specific co-evolution of the Egf receptor/ligand signaling system. BMC Evol Biol (2010) 10:27. 10.1186/1471-2148-10-27 20105326 PMC2834686

[8] Laisney JA Mueller TD Schartl M Meierjohann S. Hyperactivation of constitutively dimerized oncogenic EGF receptors by autocrine loops. Oncogene (2013) 32:2403–11. 10.1038/onc.2012.267 22751127

[9] Schartl M Wilde B Laisney JA Taniguchi Y Takeda S Meierjohann S. A mutated EGFR is sufficient to induce malignant melanoma with genetic background-dependent histopathologies. J Invest Dermatol (2010) 130:249–58. 10.1038/jid.2009.213 19609310

[10] Schartl M Kneitz S Wilde B Wagner T Henkel CV Spaink HP Conserved expression signatures between medaka and human pigment cell tumors. PLoS One (2012) 7:e37880. 10.1371/journal.pone.0037880 22693581 PMC3365055

[11] Wittbrodt J Adam D Malitschek B Maueler W Raulf F Telling A Novel putative receptor tyrosine kinase encoded by the melanoma-inducing Tu locus in *Xiphophorus*. Nature (1989) 341:415–21. 10.1038/341415a0 2797166

[12] Anders F. Contributions of the Gordon-Kosswig melanoma system to the present concept of neoplasia. Pigment Cell Res (1991) 4:7–29. 10.1111/j.1600-0749.1991.tb00309.x 1924175

[13] Schaafhausen MK Yang WJ Centanin L Wittbrodt J Bosserhoff A Fischer A Tumor angiogenesis is caused by single melanoma cells in a manner dependent on reactive oxygen species and NF-kappaB. J Cell Sci (2013) 126:3862–72. 10.1242/jcs.125021 23843609

[14] Cao Y Li CY Moeller BJ Yu D Zhao Y Dreher MR Observation of incipient tumor angiogenesis that is independent of hypoxia and hypoxia inducible factor-1 activation. Cancer Res (2005) 65:5498–505. 10.1158/0008-5472.CAN-04-4553 15994919

[15] Barnhill RL Levy MA. Regressing thin cutaneous malignant melanomas (< or =1.0 mm) are associated with angiogenesis. Am J Pathol (1993) 143:99–104. 7686347 PMC1886944

[16] Semenza GL. HIF-1 mediates metabolic responses to intratumoral hypoxia and oncogenic mutations. J Clin Invest (2013) 123:3664–71. 10.1172/JCI67230 23999440 PMC3754249

[17] Schofield CJ Ratcliffe PJ Oxygen sensing by HIF hydroxylases. Nat Rev Mol Cell Biol (2004) 5:343–54 10.1038/nrm1366 15122348

[18] Hirota K Semenza GL Regulation of angiogenesis by hypoxia-inducible factor 1. Crit Rev Oncol Hematol (2006) 59:15–26 10.1016/j.critrevonc.2005.12.003 16716598

[19] Simon MP Tournaire R Pouyssegur J. The angiopoietin-2 gene of endothelial cells is up-regulated in hypoxia by a HIF binding site located in its first intron and by the central factors GATA-2 and Ets-1. J Cell Physiol (2008) 217:809–18. 10.1002/jcp.21558 18720385

[20] Sebastia J Kieran D Breen B King MA Netteland DF Joyce D Angiogenin protects motoneurons against hypoxic injury. Cell Death Differ (2009) 16:1238–47. 10.1038/cdd.2009.52 19444281

[21] Nys K Maes H Dudek AM Agostinis P. Uncovering the role of hypoxia inducible factor-1alpha in skin carcinogenesis. Biochim Biophys Acta (2011) 1816:1–12. 10.1016/j.bbcan.2011.02.001 21338656

[22] Zbytek B Peacock DL Seagroves TN Slominski A. Putative role of HIF transcriptional activity in melanocytes and melanoma biology. Dermatoendocrinol (2013) 5:239–51. 10.4161/derm.22678 24194964 PMC3772912

[23] Bedogni B Welford SM Cassarino DS Nickoloff BJ Giaccia AJ Powell MB. The hypoxic microenvironment of the skin contributes to Akt-mediated melanocyte transformation. Cancer Cell (2005) 8:443–54. 10.1016/j.ccr.2005.11.005 16338658

[24] Giatromanolaki A Sivridis E Kouskoukis C Gatter KC Harris AL Koukourakis MI. Hypoxia-inducible factors 1alpha and 2alpha are related to vascular endothelial growth factor expression and a poorer prognosis in nodular malignant melanomas of the skin. Melanoma Res (2003) 13:493–501. 10.1097/00008390-200310000-00008 14512791

[25] Bosserhoff AK Ellmann L Kuphal S. Melanoblasts in culture as an in vitro system to determine molecular changes in melanoma. Exp Dermatol (2011) 20:435–40. 10.1111/j.1600-0625.2011.01271.x 21496114

[26] Hanna SC Krishnan B Bailey ST Moschos SJ Kuan PF Shimamura T HIF1alpha and HIF2alpha independently activate SRC to promote melanoma metastases. J Clin Invest (2013) 123:2078–93. 10.1172/JCI66715 23563312 PMC3635738

[27] Mills CN Joshi SS Niles RM. Expression and function of hypoxia inducible factor-1 alpha in human melanoma under non-hypoxic conditions. Mol Cancer (2009) 8:104. 10.1186/1476-4598-8-104 19919690 PMC2781803

[28] Kumar SM Yu H Edwards R Chen L Kazianis S Brafford P Mutant V600E BRAF increases hypoxia inducible factor-1alpha expression in melanoma. Cancer Res (2007) 67:3177–84. 10.1158/0008-5472.CAN-06-3312 17409425

[29] Li YM Zhou BP Deng J Pan Y Hay N Hung MC. A hypoxia-independent hypoxia-inducible factor-1 activation pathway induced by phosphatidylinositol-3 kinase/Akt in HER2 overexpressing cells. Cancer Res (2005) 65:3257–63. 10.1158/0008-5472.CAN-04-1284 15833858

[30] Fang J Ding M Yang L Liu LZ Jiang BH. PI3K/PTEN/AKT signaling regulates prostate tumor angiogenesis. Cell Signal (2007) 19:2487–97. 10.1016/j.cellsig.2007.07.025 17826033 PMC2094004

[31] Bian CX Shi Z Meng Q Jiang Y Liu LZ Jiang BH P70S6K 1 regulation of angiogenesis through VEGF and HIF-1alpha expression. Biochem Biophys Res Commun (2010) 398:395–9 10.1016/j.bbrc.2010.06.080 20599538 PMC2928061

[32] Spinella F Rosano L Del Duca M Di Castro V Nicotra MR Natali PG Endothelin-1 inhibits prolyl hydroxylase domain 2 to activate hypoxia-inducible factor-1alpha in melanoma cells. PLoS One (2010) 5:e11241. 10.1371/journal.pone.0011241 20574527 PMC2888584

[33] van Uden P Kenneth NS Rocha S. Regulation of hypoxia-inducible factor-1alpha by NF-kappaB. Biochem J (2008) 412:477–84. 10.1042/BJ20080476 18393939 PMC2474706

[34] Kuphal S Winklmeier A Warnecke C Bosserhoff AK. Constitutive HIF-1 activity in malignant melanoma. Eur J Cancer (2010) 46:1159–69. 10.1016/j.ejca.2010.01.031 20185296

[35] Madonna G Ullman CD Gentilcore G Palmieri G Ascierto PA. NF-kappaB as potential target in the treatment of melanoma. J Transl Med (2012) 10:53. 10.1186/1479-5876-10-53 22433222 PMC3338086

[36] Meierjohann S. Oxidative stress in melanocyte senescence and melanoma transformation. Eur J Cell Biol (2014) 93:36–41. 10.1016/j.ejcb.2013.11.005 24342719

[37] Hodgkinson CA Moore KJ Nakayama A Steingrimsson E Copeland NG Jenkins NA Mutations at the mouse microphthalmia locus are associated with defects in a gene encoding a novel basic-helix-loop-helix-zipper protein. Cell (1993) 74:395–404. 10.1016/0092-8674(93)90429-T 8343963

[38] Opdecamp K Nakayama A Nguyen MT Hodgkinson CA Pavan WJ Arnheiter H. Melanocyte development in vivo and in neural crest cell cultures: crucial dependence on the Mitf basic-helix-loop-helix-zipper transcription factor. Development (1997) 124:2377–86. 9199364 10.1242/dev.124.12.2377

[39] Hartman ML Czyz M. Pro-survival role of MITF in melanoma. J Invest Dermatol (2015) 135:352–8. 10.1038/jid.2014.319 25142731

[40] Busca R Berra E Gaggioli C Khaled M Bille K Marchetti B Hypoxia-inducible factor 1{alpha} is a new target of microphthalmia-associated transcription factor (MITF) in melanoma cells. J Cell Biol (2005) 170:49–59. 10.1083/jcb.200501067 15983061 PMC2171372

[41] Cheli Y Ohanna M Ballotti R Bertolotto C. Fifteen-year quest for microphthalmia-associated transcription factor target genes. Pigment Cell Melanoma Res (2010) 23:27–40. 10.1111/j.1755-148X.2009.00653.x 19995375

[42] Carreira S Goodall J Denat L Rodriguez M Nuciforo P Hoek KS Mitf regulation of Dia1 controls melanoma proliferation and invasiveness. Genes Dev (2006) 20:3426–39. 10.1101/gad.406406 17182868 PMC1698449

[43] Giuliano S Cheli Y Ohanna M Bonet C Beuret L Bille K Microphthalmia-associated transcription factor controls the DNA damage response and a lineage-specific senescence program in melanomas. Cancer Res (2010) 70:3813–22. 10.1158/0008-5472.CAN-09-2913 20388797

[44] Loercher AE Tank EM Delston RB Harbour JW. MITF links differentiation with cell cycle arrest in melanocytes by transcriptional activation of INK4A. J Cell Biol (2005) 168:35–40. 10.1083/jcb.200410115 15623583 PMC2171666

[45] Wellbrock C Marais R. Elevated expression of MITF counteracts B-RAF-stimulated melanocyte and melanoma cell proliferation. J Cell Biol (2005) 170:703–8. 10.1083/jcb.200505059 16129781 PMC2171350

[46] Bertolotto C Lesueur F Giuliano S Strub T De Lichy M Bille K A SUMOylation-defective MITF germline mutation predisposes to melanoma and renal carcinoma. Nature (2011) 480:94–8. 10.1038/nature10539 22012259

[47] Ryan HE Poloni M Mcnulty W Elson D Gassmann M Arbeit JM Hypoxia-inducible factor-1alpha is a positive factor in solid tumor growth. Cancer Res (2000) 60:4010–5. 10945599

[48] Yang Y Wu J Demir A Castillo-Martin M Melamed RD Zhang G GAB2 induces tumor angiogenesis in NRAS-driven melanoma. Oncogene (2013) 32:3627–37. 10.1038/onc.2012.367 22926523 PMC3813964

[49] Aguirre-Ghiso JA Frankel P Farias EF Lu Z Jiang H Olsen A RalA requirement for v-Src- and v-Ras-induced tumorigenicity and overproduction of urokinase-type plasminogen activator: involvement of metalloproteases. Oncogene (1999) 18:4718–25. 10.1038/sj.onc.1202850 10467419

[50] Diebold I Djordjevic T Petry A Hatzelmann A Tenor H Hess J Phosphodiesterase 2 mediates redox-sensitive endothelial cell proliferation and angiogenesis by thrombin via Rac1 and NADPH oxidase 2. Circ Res (2009) 104:1169–77. 10.1161/CIRCRESAHA.109.196592 19390057

[51] Craige SM Chen K Pei Y Li C Huang X Chen C NADPH oxidase 4 promotes endothelial angiogenesis through endothelial nitric oxide synthase activation. Circulation (2011) 124:731–40. 10.1161/CIRCULATIONAHA.111.030775 21788590 PMC3589548

[52] Chen JX Zeng H Lawrence ML Blackwell TS Meyrick B. Angiopoietin-1-induced angiogenesis is modulated by endothelial NADPH oxidase. Am J Physiol Heart Circ Physiol (2006) 291:H1563–72. 10.1152/ajpheart.01081.2005 16679392

[53] Abid MR Spokes KC Shih SC Aird WC. NADPH oxidase activity selectively modulates vascular endothelial growth factor signaling pathways. J Biol Chem (2007) 282:35373–85. 10.1074/jbc.M702175200 17908694

[54] Wang H Yang Z Jiang Y Hartnett ME. Endothelial NADPH oxidase 4 mediates vascular endothelial growth factor receptor 2-induced intravitreal neovascularization in a rat model of retinopathy of prematurity. Mol Vis (2014) 20:231–41. 24623966 PMC3945806

[55] Leikam C Hufnagel A Schartl M Meierjohann S. Oncogene activation in melanocytes links reactive oxygen to multinucleated phenotype and senescence. Oncogene (2008) 27:7070–82. 10.1038/onc.2008.323 18806824

[56] Leikam C Hufnagel A Walz S Kneitz S Fekete A Muller MJ Cystathionase mediates senescence evasion in melanocytes and melanoma cells. Oncogene (2014) 33:771–82. 10.1038/onc.2012.641 23353821

[57] Lokaj K Meierjohann S Schutz C Teutschbein J Schartl M Sickmann A. Quantitative differential proteome analysis in an animal model for human melanoma. J Proteome Res (2009) 8:1818–27. 10.1021/pr800578a 19249851

[58] Huang S Deguzman A Bucana CD Fidler IJ. Nuclear factor-kappaB activity correlates with growth, angiogenesis, and metastasis of human melanoma cells in nude mice. Clin Cancer Res (2000) 6:2573–81. 10873114

[59] Gao K Dai DL Martinka M Li G. Prognostic significance of nuclear factor-kappaB p105/p50 in human melanoma and its role in cell migration. Cancer Res (2006) 66:8382–8. 10.1158/0008-5472.CAN-05-4402 16951147

[60] Karst AM Gao K Nelson CC Li G. Nuclear factor kappa B subunit p50 promotes melanoma angiogenesis by upregulating interleukin-6 expression. Int J Cancer (2009) 124:494–501. 10.1002/ijc.23973 18942706

[61] Wani AA Jafarnejad SM Zhou J Li G. Integrin-linked kinase regulates melanoma angiogenesis by activating NF-kappaB/interleukin-6 signaling pathway. Oncogene (2011) 30:2778–88. 10.1038/onc.2010.644 21278793

[62] Li J Cheng Y Tai D Martinka M Welch DR Li G. Prognostic significance of BRMS1 expression in human melanoma and its role in tumor angiogenesis. Oncogene (2011) 30:896–906. 10.1038/onc.2010.470 20935672 PMC3235331

[63] Okuno Y Nakamura-Ishizu A Otsu K Suda T Kubota Y. Pathological neoangiogenesis depends on oxidative stress regulation by ATM. Nat Med (2012) 18:1208–16. 10.1038/nm.2846 22797809

[64] Ma Q Role of nrf2 in oxidative stress and toxicity. Annu Rev Pharmacol Toxicol (2013) 53:401–26 10.1146/annurev-pharmtox-011112-140320 23294312 PMC4680839

[65] Afonyushkin T Oskolkova OV Philippova M Resink TJ Erne P Binder BR Oxidized phospholipids regulate expression of ATF4 and VEGF in endothelial cells via NRF2-dependent mechanism: novel point of convergence between electrophilic and unfolded protein stress pathways. Arterioscler Thromb Vasc Biol (2010) 30:1007–13 10.1161/ATVBAHA.110.204354 20185790

[66] Afonyushkin T Oskolkova OV Binder BR Bochkov VN. Involvement of CK2 in activation of electrophilic genes in endothelial cells by oxidized phospholipids. J Lipid Res (2011) 52:98–103. 10.1194/jlr.M009480 20934988 PMC2999922

[67] Kim TH Hur EG Kang SJ Kim JA Thapa D Lee YM NRF2 blockade suppresses colon tumor angiogenesis by inhibiting hypoxia-induced activation of HIF-1alpha. Cancer Res (2011) 71:2260–75. 10.1158/0008-5472.CAN-10-3007 21278237

[68] Zhang Z Wang Q Ma J Yi X Zhu Y Xi X Reactive oxygen species regulate FSH-induced expression of vascular endothelial growth factor via Nrf2 and HIF1alpha signaling in human epithelial ovarian cancer. Oncol Rep (2013) 29:1429–34. 10.3892/or.2013.2278 23404377

[69] Ji X Wang H Zhu J Zhu L Pan H Li W Knockdown of Nrf2 suppresses glioblastoma angiogenesis by inhibiting hypoxia-induced activation of HIF-1alpha. Int J Cancer (2014) 135:574–84. 10.1002/ijc.28699 24374745

[70] Guzy RD Schumacker PT. Oxygen sensing by mitochondria at complex III: the paradox of increased reactive oxygen species during hypoxia. Exp Physiol (2006) 91:807–19. 10.1113/expphysiol.2006.033506 16857720

[71] Calvani M Comito G Giannoni E Chiarugi P. Time-dependent stabilization of hypoxia inducible factor-1alpha by different intracellular sources of reactive oxygen species. PLoS One (2012) 7:e38388. 10.1371/journal.pone.0038388 23144690 PMC3483303

[72] Maniotis AJ Folberg R Hess A Seftor EA Gardner LM Pe’er J Vascular channel formation by human melanoma cells in vivo and in vitro: vasculogenic mimicry. Am J Pathol (1999) 155:739–52. 10.1016/S0002-9440(10)65173-5 10487832 PMC1866899

[73] Thies A Mangold U Moll I Schumacher U. PAS-positive loops and networks as a prognostic indicator in cutaneous malignant melanoma. J Pathol (2001) 195:537–42. 10.1002/path.988 11745688

[74] Vartanian AA Burova OS Stepanova EV Baryshnikov AY Lichinitser MR. Melanoma vasculogenic mimicry is strongly related to reactive oxygen species level. Melanoma Res (2007) 17:370–9. 10.1097/CMR.0b013e3282f1d2ec 17992120

[75] Biswas SK Lewis CE. NF-kappaB as a central regulator of macrophage function in tumors. J Leukoc Biol (2010) 88:877–84. 10.1189/jlb.0310153 20573802

[76] Yan L Yee JA Mcguire MH Graef GL. Effect of dietary supplementation of selenite on pulmonary metastasis of melanoma cells in mice. Nutr Cancer (1997) 28:165–9. 10.1080/01635589709514570 9290123

[77] Yan L Yee JA Li D Mcguire MH Graef GL. Dietary supplementation of selenomethionine reduces metastasis of melanoma cells in mice. Anticancer Res (1999) 19:1337–42. 10368696

[78] Tosetti F Ferrari N De Flora S Albini A Angioprevention: angiogenesis is a common and key target for cancer chemopreventive agents. FASEB J (2002) 16:2–14 10.1096/fj.01-0300rev 11772931

[79] Wang M Kaufman RJ. The impact of the endoplasmic reticulum protein-folding environment on cancer development. Nat Rev Cancer (2014) 14:581–97. 10.1038/nrc3800 25145482

[80] Yadav RK Chae SW Kim HR Chae HJ Endoplasmic reticulum stress and cancer. J Cancer Prev (2014) 19:75–88 10.15430/JCP.2014.19.2.75 25337575 PMC4204165

[81] Wang Y Alam GN Ning Y Visioli F Dong Z Nor JE The unfolded protein response induces the angiogenic switch in human tumor cells through the PERK/ATF4 pathway. Cancer Res (2012) 72:5396–406. 10.1158/0008-5472.CAN-12-0474 22915762 PMC3743425

[82] Pereira ER Liao N Neale GA Hendershot LM. Transcriptional and post-transcriptional regulation of proangiogenic factors by the unfolded protein response. PLoS One (2010) 5:e12521. 10.1371/journal.pone.0012521 20824063 PMC2932741

[83] Zhuang L Scolyer RA Lee CS Mccarthy SW Cooper WA Zhang XD Expression of glucose-regulated stress protein GRP78 is related to progression of melanoma. Histopathology (2009) 54:462–70. 10.1111/j.1365-2559.2009.03242.x 19309398

[84] Xue Y Petrovic N Cao R Larsson O Lim S Chen S Hypoxia-independent angiogenesis in adipose tissues during cold acclimation. Cell Metab (2009) 9:99–109. 10.1016/j.cmet.2008.11.009 19117550

[85] Arany Z Foo SY Ma Y Ruas JL Bommi-Reddy A Girnun G HIF-independent regulation of VEGF and angiogenesis by the transcriptional coactivator PGC-1alpha. Nature (2008) 451:1008–12. 10.1038/nature06613 18288196

[86] Vazquez F Lim JH Chim H Bhalla K Girnun G Pierce K PGC1alpha expression defines a subset of human melanoma tumors with increased mitochondrial capacity and resistance to oxidative stress. Cancer Cell (2013) 23:287–301. 10.1016/j.ccr.2012.11.020 23416000 PMC3708305

[87] Haq R Shoag J Andreu-Perez P Yokoyama S Edelman H Rowe GC Oncogenic BRAF regulates oxidative metabolism via PGC1alpha and MITF. Cancer Cell (2013) 23:302–15. 10.1016/j.ccr.2013.02.003 23477830 PMC3635826

[88] Damsky WE Theodosakis N Bosenberg M. Melanoma metastasis: new concepts and evolving paradigms. Oncogene (2014) 33:2413–22. 10.1038/onc.2013.194 23728340

[89] Ulmer A Dietz K Hodak I Polzer B Scheitler S Yildiz M Quantitative measurement of melanoma spread in sentinel lymph nodes and survival. PLoS Med (2014) 11:e1001604. 10.1371/journal.pmed.1001604 24558354 PMC3928050

